# Test for Glycoronic Acid

**Published:** 1914-06

**Authors:** 


**Test for Glycoronic Acid.** To 10 c.c. of urine, are added 2 c.c. of dilute sulphuric or phosphoric acid, 10 of grain alcohol, 20 of ether. Shake. Separate and evaporate the ether-alcohol extracts which contains the glycorunonic acid if present. Test the residue with orcin-naphtho-resorcin. O. Schweket, Biochem. Zeitschrift, No. 1 and 2, Vol. 55.

